# The Bax inhibitor MrBI-1 regulates heat tolerance, apoptotic-like cell death, and virulence in *Metarhizium robertsii*

**DOI:** 10.1038/srep10625

**Published:** 2015-05-29

**Authors:** Yixiong Chen, Zhibing Duan, Peilin Chen, Yanfang Shang, Chengshu Wang

**Affiliations:** 1 Key Laboratory of Insect Developmental and Evolutionary Biology, Institute of Plant Physiology and Ecology, Shanghai Institutes for Biological Sciences, Chinese Academy of Sciences, Shanghai 200032, China; 2 Current address:Department of Neuroscience & Cell Biology, Robert Wood Johnson Medical School, Rutgers University, Piscataway NJ, 08854, USA

## Abstract

Bax inhibitor 1 (BI-1) is a highly conserved protein originally identified as a suppressor of the proapoptotic protein Bax to inhibit cell death in animals and plants. The orthologs of BI-1 are widely distributed in filamentous fungi but their functions remain largely unknown. Herein, we report the identification and characterizations of *MrBI-1*, an ortholog of BI-1, in the entomopathogenic fungus *Metarhizium robertsii*. First, we found that MrBI-1 could partially rescue mammalian Bax-induced cell death in yeast. Deletion of *MrBI-1* impaired fungal development, virulence and heat tolerance in *M. robertsii*. We also demonstrated that inactivation of *MrBI-1* reduced fungal resistance to farnesol but not to hydrogen peroxide, suggesting that MrBI-1 contributes to antiapoptotic-like cell death via the endoplasmic reticulum stress-signaling pathway rather than the classical mitochondrium-dependent pathway. In particular, we found that unlike the observations in yeasts and plants, expression of mammalian Bax did not lead to a lethal effect in *M. robertsii*; however, it did aggravate the fungal apoptotic effect of farnesol. The results of this study advance our understanding of BI-1-like protein functions in filamentous fungi.

Programmed cell death (PCD) is a genetically controlled and highly conserved process occurring in different organisms and include two categories, i.e. apoptosis (type I PCD) and autophagy (type II PCD)^1^. Both types have been well documented in fungi^2^. For cellular apoptosis, the mitochondrial (MT) pathways with the typical feature of cytochrome *c* release have been described in different filamentous fungi such as *Aspergillus nidulans*^3^,^4^,^5^,^6^, *Neurospora crassa*^7^, and *Fusarium graminearum*^8^. The identifications and subsequent characterizations of Bcl-2 family members, e.g. Bcl-2, Bax, Bak, etc., in animals revealed the endoplasmic reticulum (ER) pathways of apoptosis^9^. Bcl-2 family proteins are not present in fungi; however, exogenous expression of proapoptotic factor Bax or Bak could induce typical apoptotic characteristics, whereas the expression of antiapoptotic factor Bcl-2 could inhibit cell death in both budding and fission yeasts^10^,^11^, indicating the presence of conserved apoptotic machineries in fungi.

Bax inhibitor-1 (BI-1) was first identified in mammals but functionally verified in yeast and shown to suppress Bax-induced cell death^10^. Unlike the Bcl-2 family members, BI-1 is evolutionarily conserved and widely present in eukaryotic species from fungi to plants and animals^12^. BI-1 proteins of different organisms have either six or seven transmembrane domains and are predominantly localized to the ER membrane^10^,^13^. It has been demonstrated that BI-1 can interact with Bcl-2 family members so as to enable the cells to adapt to a wide range of stresses via ER-signaling pathways^14^,^15^,^16^. Homologs of BI-1 have also been cloned and characterized in plants, e.g. AtBI-1 from *Arabidopsis*^17^,^18^,^19^ and BXI1 from the budding yeast *Saccharomyces cerevisiae*^20^. AtBI-1 can interact with different proteins to activate the hypersensitive response with PCD-type plant epidermal cell death against fungal pathogen infections^21^,^22^. Yeast BXI1 is an ER-localized protein and the gene deletion mutant has become more sensitive to ER-stress drugs^20^. Unlike fungi, plants encode a family of ER-residing Bag (Bcl-2 associated anthogene) proteins that can interact with different proteins to regulate the apoptosis-like processes ranging from abiotic stresses to pathogen attacks^23^. Prior to this study, the distribution and function(s) of BI-1 homologs remained unknown in filamentous fungi.

Ascomycete insect pathogenic fungi such as *Metarhizium* spp. diverged after the yeasts^24^, and these species have been studied as model organisms for underpinning the mechanisms of insect-fungus interactions^25^,^26^. Various virulence-related genes have been functionally characterized, including the autophagy-related genes^27^,^28^. Oxidative stress related MT-signaling pathways have been evident with an apoptosis-like aging in *Metarhizium* species^3^,^29^,^30^. In the present work, an ortholog of yeast *BXI1* gene, designated *MrBI-1* (MAA_10304, 30% identity at the protein sequence level), was identified and deleted in *M. robertsii*. We found that MrBI-1 partially rescued Bax-induced lethal effects in yeasts, and that the protein contributed to heat tolerance and antiapoptotic-like cell death in *M. robertsii*. Deletion of *MrBI-1* also impaired fungal virulence.

## Results

### Phylogeny and secondary structure analyses

**Figure 1:**
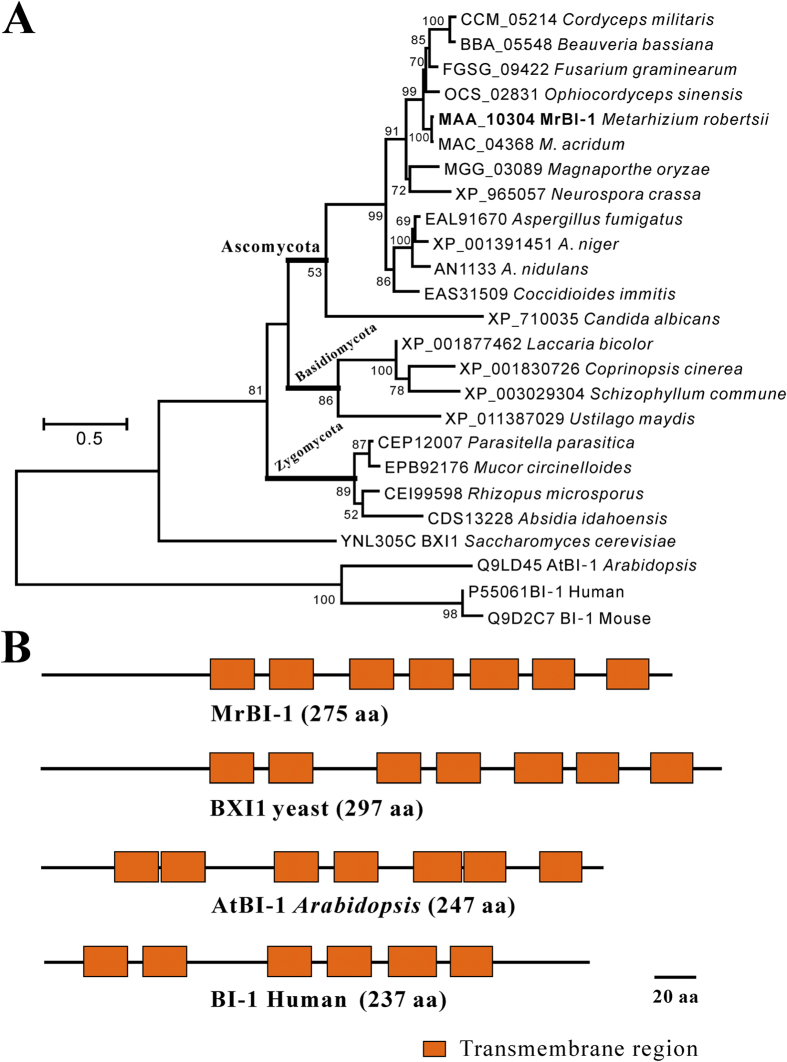
Phylogenetic analysis and transmembrane domain comparison. (**A**) Phylogenetic analysis of MrBI-1 (highlighted in bold) with different homologs. The protein sequences were aligned with CLUSTAL X and a Maximum likelihood tree was generated using a Dayhoff substitution model. (**B**) Schematic comparison of transmembrane domains present in MrBI-1, yeast BXI1, *Arabidopsis* AtBI-1 and human BI-1.

Genome-wide surveys of different ascomycete fungal genomes indicated that as in mammals, plants, and yeasts, a single copy of a *BI-1*-like gene is present in *M. robertsii* and other ascomycetous, basidiomycetous and zygomycetous fungi. The extensive genome surveys indicated that the BI-1-like proteins are not present in the basal fungal genomes of chytrids and microsporidians. A phylogenetic analysis demonstrated that MrBI-1 is mostly closely related to the homolog (MAC_04368) from *M. acridum* (a locust-specific pathogen), and that it was then clustered with those from hypocrealean fungi (Fig. 1A). In general, except for the yeast BXI1, the phylogeny of BI-1 proteins is congruent with organism speciation^24^, an indicator of a highly conserved relationship among these orthologs. Scanning of the transmembrane domain revealed that, in contrast to human BI-1, MrBI-1 contains seven transmembrane domains like the orthologs from yeast (BXI1) and *Arabidopsis* (AtBI-1) (Fig. 1B).

### MrBI-1 partially rescued the Bax-induced growth defect in yeast

**Figure 2:**
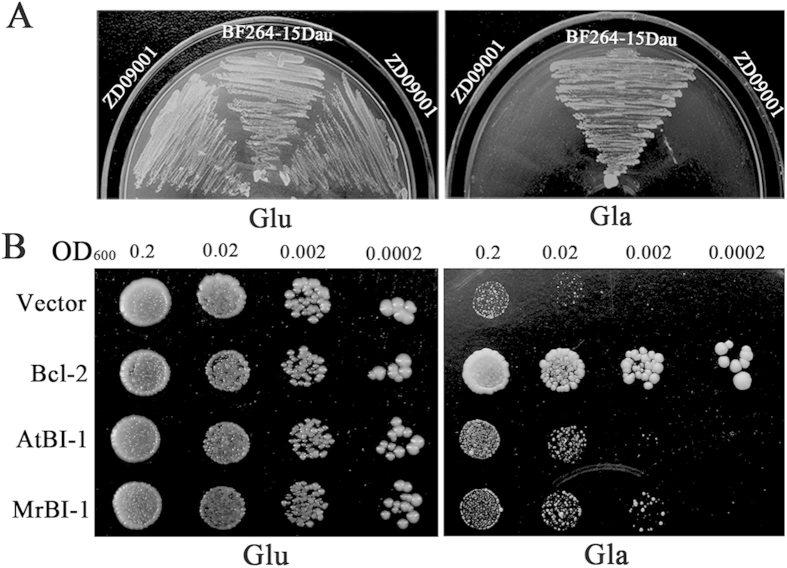
Functional assay of MrBI-1 in yeasts. (**A**) Yeast strain BF264-15Dau was transformed with the vector pTS909-*Bax* and the resultant strain ZD09001 was either streaked on a SD-Trp (Glu) or on an SD-Trp (Gal) plate, and incubated at 30 °C for 3 d. Glu, glucose; Gal, galactose. All strains grew equally well on a SD-Trp (Glu) plate while the ZD09001 cells lost their viability when streaked on an SD-Trp (Gal) plate. (**B**) ZD09001 was transformed by various vectors (pYX112-*Bcl2*, pYX112-*AtBI,* and pYX112-*MrBI*, pYX112 as control). Yeast strains of 5 μl by gradient were spotted on either SD-Trp-Ura (Glu) or SD-Trp-Ura (Gal) plates and incubated at 30 °C for 3 d. The viabilities of the yeast cells containing *Bcl-2*, *MrBI-1* and *AtBI-1* genes were increased to varied degrees when compared with the control strain transformed with the empty vector.

The yeast strains harboring either an empty vector or the plasmid containing a galactose-inducible promoter-controlled *Bax* gene, i.e. the ZD09001 strain, grew equally well on a glucose-containing synthetic drop-out (SD) medium. However, once induced on a galactose medium, ZD09001 cells lost their viability due to the lethal effect of Bax (Fig. 2A). To determine whether MrBI-1 could suppress Bax-induced cell death in yeast, the cDNA of *MrBI-1* as well as the positive controls of mammalian *Bcl-2* and *Arabidopsis AtBI-1* genes were used for yeast transformations. All yeast strains obtained grew equally well on the glucose medium. However, once Bax protein expression was induced by galactose, in contrast to the negative control, the Bcl-2-containing strain showed complete viability; while similar to the *AtBI-1 -*transformed strain, *MrBI-1* partially suppressed Bax-induced cell death in yeast (Fig. 2B).

### Gene deletion and phenotypic characterization

**Figure 3:**
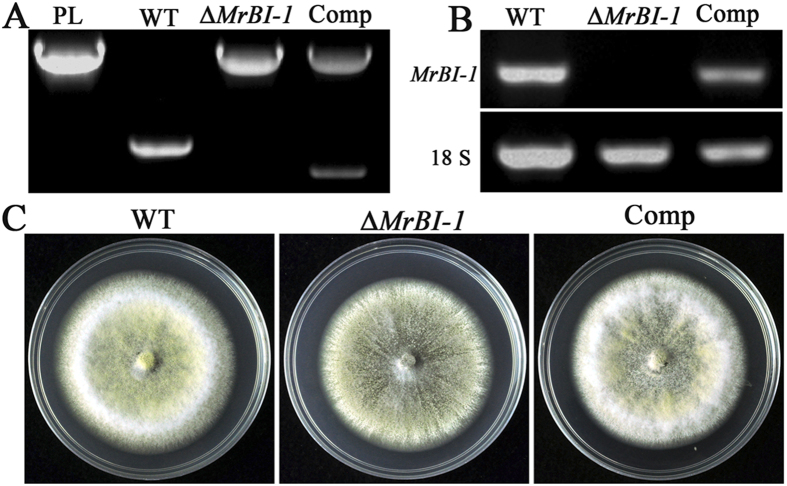
Verification of fungal transformation and phenotypic characterization. (**A**) PCR verification. The plasmid (PL) pBar*MrBI* was used as a positive control. (**B**) RT-PCR verification to confirm the loss and recovery of *MrBI-1* transcripts. The small subunit ribosomal gene (18 S rRNA) was used as a reference. (**C**) Growth characteristics of WT, Δ*MrBI-1,* and Comp on PDA for 15 d. No growth-rate variation was observed among the WT, *ΔMrBI-1* and comp.

**Figure 4:**
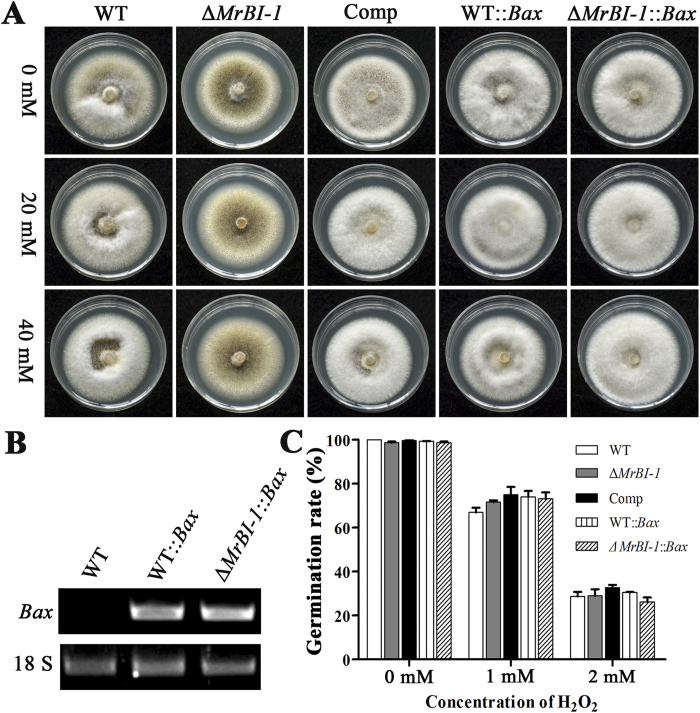
Growth test and spore survival assays against H_2_O_2_ challenge. (**A**) Phenotypes of the WT, *ΔMrBI-1*, Comp, WT::*Bax* and *ΔMrBI-1*::*Bax* on PDA or PDA supplemented with 20 or 40 mM H_2_O_2_ for 10 d. No obvious difference in growth rate was observed between the WT and mutants. (**B**) Verification of exogenous *Bax* gene expression. The strains were grown in SDB for 3 d and used for RT-PCR analysis to verify the transcription of *Bax* gene in WT::*Bax* and *ΔMrBI-1*::*Bax*. The 18 S rRNA gene was used as a reference. (**C**) Germination rates of different strain conidia in the presence of H_2_O_2_. The conidia of the WT, Δ*MrBI-1*, Comp, WT::*Bax* and *ΔMrBI-1*::*Bax* were suspended in SDB with 1 or 2 mM H_2_O_2_ for 12 h.

To investigate the potential function of MrBI-1 in *M. robertsii*, the gene was deleted by homologous recombination. Gene complementation was performed by transformation of *ΔMrBI-1* with the *MrBI-1* cDNA to obtain the mutant Comp. Genetically stable transformants were verified by PCR (Fig. 3A) and RT-PCR (Fig. 3B). In terms of the growth rate, no obvious difference was observed when growing the wild-type (WT) and mutants on potato dextrose agar (PDA) (Figs. 3C and 4A). As indicated above, the proapoptotic Bax-like factor is not present in fungi or plants^31^. To further determine the function of MrBI-1, the mammalian *Bax* gene was made under the control of a constitutive promoter and used to transform the WT and *ΔMrBI-1* strains. The successfully obtained transformants were designated WT*::Bax* and *ΔMrBI-1::Bax*, respectively. When grown on PDA, no growth rate variations were observed for these mutants when compared to the WT of *M. robertsii* (Fig. 4A). Our RT-PCR analysis confirmed that the exogenous *Bax* gene could be similarly expressed by the fungi (Fig. 4B).

### Deletion of *MrBI-1* increased fungal sensitivity to heat shock but not to H_2_O_2_

**Figure 5:**
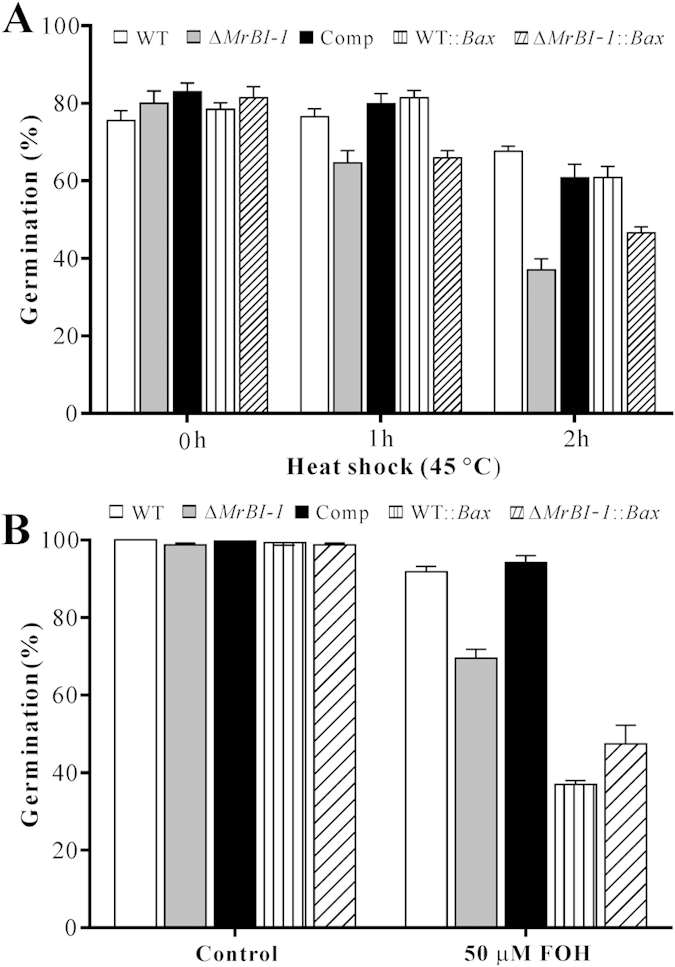
Spore survival assays against heat shock and FOH challenges. (**A**) Spore germinations after heat shock treatment. Conidia of the WT, Δ*MrBI-1*, Comp, WT::*Bax* and Δ*MrBI-1*::*Bax* were treated at 45 °C in a water bath for different time periods, and inoculated on PDA at 25 °C for 24 h. Conidial survival rate of different mutants varied to different degrees when compared with the WT. (**B**) Spore germinations in the presence of FOH. The conidia of the WT, Δ*MrBI-1*, Comp, WT::*Bax* and Δ*MrBI-1*::*Bax* were suspended in SDB with 50 μM FOH at 25 °C for 16 h. Conidial survival rate of different mutants varied to different degrees when compared with the WT.

To examine the effect of MrBI-1 on different stress factors, the spores of the WT and mutants were challenged with H_2_O_2_ or heat shock. The growth of mutants was not inhibited when the fungi were grown on PDA or PDA supplemented with H_2_O_2_ as compared to the WT of *M. robertsii* (Fig. 4A). During germination assays, the survival rates of the WT and mutant spores were equally reduced after the treatment with H_2_O_2_, i.e. the viabilities of mutant spores were not significantly different from those of the WT (Fig. 4C). This is consistent with the report in yeasts that BXI1 is not involved in suppressing H_2_O_2_-induced cell death^12^. Heat shock treatment indicated, however, that significant differences in conidial survival rates were observed between the WT and mutants after exposure to 45 °C for 1 or 2 h (Fig. 5A). For example, the viabilities of *ΔMrBI-1* spores were significantly reduced when treated either for 1 h (*t-*test, *P* = 0.0311) or 2 h (*P* = 0.0038) when compared with the WT. In addition, relative to the WT, overexpression of *Bax* in *ΔMrBI-1* but not in the WT further increased fungal sensitivity to heat shock (*P* = 0.0238 for treatment for 1 h; *P* = 0.0072 for 2 h). The differences of spore survival were not significant between *ΔMrBI-1* and *ΔMrBI-1::Bax* after exposure to 45 °C for 1 (*P* = 0.2152) but significant after exposure for 2 h (*P* = 0.0282) (Fig. 5A). These observations thereby indicated that MrBI-1 was not involved in the oxidative stress response but in heat tolerance in *M. robertsii*.

### MrBI-1 contributes to antiapoptotic-like cell death in *M. robertsii*

**Figure 6:**
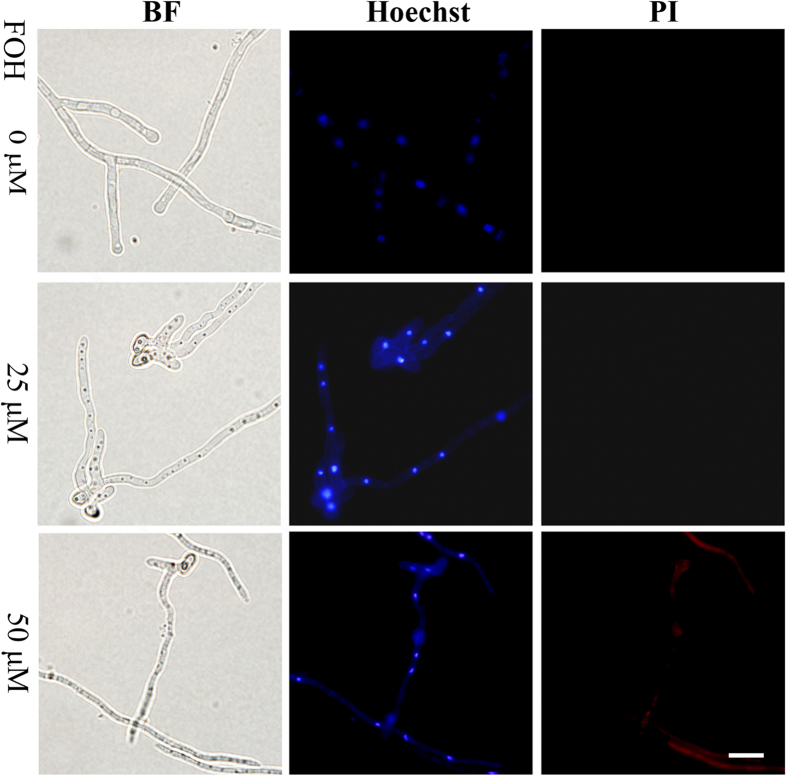
Apoptosis induction in *M. robertsii*. Germlings of the WT were treated with or without FOH (25 or 50 μM) at 25 °C for 4 h and double stained with Hoechst 33342/PI dyes. Bright field (BF) optics showed a dose-dependent inhibition of hyphal growth. The cells treated with 25 μM FOH displayed the apoptotic characteristic of chromatin condensation and marginalization characteristic of apoptosis, whereas the germlings treated with 50 μM FOH were infiltrated by PI, indicative of cell necrosis. Bar = 10 μm.

To determine whether MrBI-1 contributed to the suppression of apoptotic cell death in *M. robertsii*, fungal cells were treated with the apoptosis-inducing compound farnesol (FOH)^32^. After exposure to 25 or 50 μM FOH for 4 h, the WT germlings were co-stained with Hoechst and propidium iodide (PI) dyes. The observations revealed the apoptotic features of intense chromatin condensation and marginalization in the cells treated with 25 μM FOH. The exposure to 50 μM FOH even triggered cell necrosis as revealed by PI staining signals (Fig. 6). Based on these results, the contribution of MrBI-1 to the suppression of FOH-induced apoptotic-like cell death was evaluated by inoculation of the WT and different mutant spores in Sabouraud dextrose broth (SDB) in the presence or absence of FOH (50 μM) for 16 h (Fig. 5B). When compared with the WT (91.8%), spore germination rates of *ΔMrBI-1* (69.5%, *P* = 0.0111), WT*::Bax* (36.9%, *P* = 0.0007), and *ΔMrBI-1::Bax* (47.4%, *P* = 0.0035) were significantly reduced. No significant difference was observed between WT and Comp. The differences between the WT and WT*::Bax* (*P* = 0.0007), and between the *ΔMrBI-1* and *ΔMrBI-1::Bax* (*P* = 0.0285) were also significant; however, there was no significant difference between WT*::Bax* and Δ*MrBI-1::Bax* (*P* = 0.1019) (Fig. 5B).

### MrBI-1 is required for full virulence in *M. robertsii*

To investigate the effects of *MrBI-1* deletion on fungal virulence, insect bioassays were conducted against silkworm larvae. The median lethal time (LT_50_) values were estimated and compared among the WT, *ΔMrBI-1* and Comp. The results indicated that the differences between the WT (LT_50_ = 2.5 ± 0.093 days) and *ΔMrBI-1* (LT_50_ = 3.0 ± 0.284 days), and between *ΔMrBI-1*and Comp (LT_50_ = 2.5 ± 0.186 days) were significant (*P* < 0.05); but that this was not the case between the WT and Comp. The results thereby indicated that deletion of *MrBI-1* impaired full virulence in *M. robertsii*.

## Discussion

In the present study, we characterized a *bona fide* homolog of BI-1, MrBI-1, in the insect pathogenic fungus *M. robertsii*. Not surprisingly, MrBI-1 was able to suppress Bax-induced cell death in yeast to some extent. Deletion of *MrBI-1* revealed that MrBI-1 is required for fungal heat tolerance, full virulence, and contributes to ER-stress inducer FOH, but not to the MT-sensitive factor H_2_O_2_. Human BI-1 was first identified as being able to rescue yeast cells containing the proapoptotic factor Bax^10^. Those experiments demonstrated that the endogenous BI-1 gene (i.e. *BXI1*) of yeast could not counteract the apoptotic effect caused by mammalian Bax. In support of this, our experiments indicated that unlike Bcl-2, both MrBI-1 and AtBI-1 could only partially rescue *Bax*-transformed yeast cells, even when integrated with *BXI1*’s function (Fig. 2B). Given that the orthologs of BI-1 are highly conserved (Fig. 1), the results of protein interaction assays revealed that the *Arabidopsis* AtBI-1 interacted with cytochrome *b*_*5*_ in plants, but interacted with a fatty acid hydroxylase in yeast to mediate the hydroxylation of fatty acids^33^. This would explain much of why neither AtBI-1 nor MrBI-1 failed to completely inhibit the apoptotic effect of Bax, as occurs with Bcl-2 in yeast.

FOH is a 15-carbon isoprenoid alcohol that is widely distributed in nature as an odoriferous compound and acts as a precursor in the isoprenoid/sterol biosynthetic pathway^34^. FOH has been reported to induce apoptosis-like PCD in different fungal species, e.g. *S. cerevisiae*, *A. nidulans,* and *Candida albicans*^10^. In the present study, we established that FOH triggered cellular apoptosis and even necrosis at a higher concentration in *M. robertsii*. Observations in mammalian cells indicated that deletion of *BI-1* did not increase cell sensitivity to MT stress, but did against ER-stress agents^35^,^36^. Consistent with this, our results showed that MrBI-1 was not involved in H_2_O_2_-induced growth defects or cell death in *M. robertsii* (Fig. 4). Thus, we assert that MrBI-1 contributes to antiapoptotic effects in *M. robertsii* via an ER but not MT stress-response pathway. However, the exact mechanism remains to be elucidated.

Experiments in yeasts^11^,^33^ and plant cells^37^ indicated that heterologous expression of mammalian Bax could result in cell death even with the presence of endogenous BI-1 genes. This was also evident in the filamentous plant pathogenic fungus *Colletotrichum gloeosporioides*^38^. In contrast (and unexpectedly), expression of Bax did not induce cell death in either the WT or *ΔMrBI-1* of *M. robertsii* (Fig. 4A). In animal cells, Bax forms a homodimer, multidimer or heterodimer with Bcl-2, while BI-1 can interact with Bcl-2 but not Bax or Bak^10^,^39^. Co-expression of Bcl-2 and Bax in *C. gloeosporioides* did not lead to cell death^38^. The lack of Bax and Bcl-2 in fungal and plant cells suggests that the heterodimer of Bax and Bcl-2 could not be formed in these heterologous systems. Thus, the disparate non-lethal effect of Bax in *Metarhizium* suggests that much, if not all, of the aggregation status of Bax varied, and thereby played distinct roles in different filamentous fungi, yeasts, and plant cells. Consistent with a non-lethal effect, the heat-tolerant abilities of WT*::Bax* and *ΔMrBI-1::Bax* mutants were not reduced when compared with either the WT or *ΔMrBI-1* (Fig. 5A). However, relative to the WT and *ΔMrBI-1*, the germination rates of WT*::Bax* and *ΔMrBI-1::Bax* spores were significantly reduced when exposed to FOH (Fig. 5B), indicating that Bax aggravated the apoptotic effect in *M. robertsii* under the stress agent. Different from the yeast assay, the erratic results between the heat shock and FOH induction would suggest that MrBI-1 might not fully function via a Bax-inhibitor mechanism on the basis that Bax is evolutionarily missing in *M. robertsii*. For example, the spores of Δ*MrBI-1::Bax* had a higher (*P* = 0.0282) survival rate than those of Δ*MrBI-1* under heat shock for 2 h, while the spore survival rates between the WT*::Bax* and Δ*MrBI-1::Bax* were significantly different when the fungi were exposed to heat shock for 2 h (*P* = 0.0095) but not to FOH (*P* = 0.1019) (Fig. 5). The exact mechanism(s) involved remains to be elucidated in terms of the disparity of the Bax effects on cell viability between *M. robertsii* and yeasts or plants.

Pathogenic infections can trigger a hypersensitive response (HR) in plants, including the generation of an oxidative burst that is toxic to both pathogen and plant cells, and thereby the rapid cell death in the region surrounding an infection^40^. In *Arabidopsis*, AtBI-1 has been functionally implicated in the regulation of cell death in response to fungal infection^22^. These pathogens, however, secrete avirulence effector(s) and other pathogenicity-related proteins to counteract plant PCD-like immunities so as to allow for successful infection^41^. An HR-like response of melanization and cell death also occurs in insects when infected by *M. robertsii* during cuticle penetration^42^. After reaching the insect hemocoel, fungal pathogens such as *Metarhizium* species have to adapt to osmotic stress in hemolymph^43^, and counteract antifungal effects imposed by host cellular and humoral immune responses^26^. Thus, the attenuated virulence of *ΔMrBI-1* would then be expected to be due to its reduced resistance to cell death triggered by insect immune reactions when compared to the WT.

In conclusion, we herein report the function of MrBI-1 in an insect pathogenic fungus *M. robertsii*. Heterologous expression of MrBI-1 partially suppressed the lethal effect of mammalian Bax in yeast. In contrast to the observations in yeast and plant cells, Bax could not induce cell death in *M. robertsii*. Our data indicate for the first time that MrBI-1 is involved in ER-related apoptosis-like PCD, which contributes to fungal development and virulence.

## Methods

### Fungal strains and growth conditions

The WT strain and transformants of *M. robertsii* ARSEF 2575 (previously classified as *M. anisopliae*) were routinely cultured on PDA (Difco) at 25 °C. For liquid incubation, fungi were grown in SDB (Difco) at 25 °C in a rotatory shaker. Conidium suspensions were prepared in 0.05% (v/v) Tween-80 and filtered through four layers of sterile lens-cleaning tissues to remove hyphal fragments. Yeast strains used in this study were cultured on different media including yeast extract peptone dextrose (YPD, 1% yeast extract, 2% peptone, and 2% glucose), YPDA (YPD plus 0.008% adenine), and synthetic drop-out medium (0.17% yeast nitrogen base, amino acids and different carbon sources, with or without urea).

### Phylogenetic and transmembrane domain analyses

To establish the phylogenetic relationship between MrBI-1 and its orthologs, orthologous protein sequences from representative fungal species and those of mouse, human, and *A. thaliana* were aligned using the program CLUSTAL X^44^. A Maximum likelihood tree was generated using the program MEGA (ver. 6.0)^45^ with a Dayhoff substitution model, a Nearest-Neighbor-Interchange heuristic method for tree inference, 1,000 bootstrap replications for phylogeny test, and a partial deletion for gaps/missing data. Transmembrane domains of MrBI-1, yeast BXI1, *Arabidopsis* AtBI-1, and human BI-1 were analyzed on the TMHMM server v. 2.0 ( http://www.cbs.dtu.dk/services/TMHMM/).

### Gene function assays in yeasts

The coding region of the mammalian apoptotic gene *Bax* from the rat *Rattus norvegicus* was introduced into a *Hind*III/*Sal*I–digested yeast expression vector pTS909 under the control of the *GAL1* promoter^33^. The resultant vector pTS909-Bax was used to transform the yeast strain BF264-15Dau using a lithium acetate method^46^. The resultant strain, named ZD09001, was maintained in a SD medium lacking tryptophan (SD-Trp). The cDNA of *MrBI*-1 was cloned into the yeast vector pYX112 under the control of the triosephosphate isomerase gene (*TPI*) promoter to produce pYX112-*MrBI*. The coding regions of the *Bax* inhibitor gene *Bcl-2* from *R. norvegicus*^47^ and *AtBI-1* from the plant *A. thaliana*^13^ were cloned and used to generate the plasmids pYX112-Bcl-2 and pYX112-AtBI, respectively. These three plasmids (pYX112-MrBI, pYX112-*Bcl-2*, and pYX112-AtBI1) and the control vector pYX112 were transformed into the yeast strain ZD09001. Transformants were spotted either on an SD-glucose plate or on an SD-galactose plate, and incubated at 30 °C for 3 d.

### Gene deletion and complementation

Targeted gene deletion of *MrBI-1* gene was performed by homologous recombination as we described previously^48^. Briefly, the two primer pairs MrBI1U/MrBI1L and MrBI2U/MrBI2L (Table 1) were used to amplify the 5′- and 3′-flanking regions of the target gene *MrBI-1*, respectively. The PCR products were digested with the restriction enzymes *BamH*I and *Xba*I, respectively, and then inserted into the corresponding sites of the binary vector pDHt-bar (conferring resistance against ammonium glufosinate) to generate the plasmid pBarMrBI for *Agrobacterium*-mediated fungal transformation (ATMT). For gene complementation, the binary vector pBenMrBI was constructed by inserting the full cDNA sequence of *MrBI-1* into the plasmid pDHt-ben-gpdA (conferring resistance against Benomyl) under control of the *Aspergillus nidulans* GPDA promoter (gpdA)^27^, and transformed into the *MrBI-1* null mutant by ATMT to generate the complementary mutant (Comp). For ectopic expression of Bax in *M. robertsii*, the full-length cDNA of murine *Bax* gene was amplified by PCR with the primers BaxF and BaxR using the plasmid pTS909-Bax DNA as a template. The purified fragment was cloned into the *EcoR*I site of the pGPDBen vector to yield the pGPDBen-Bax expression vector, which was then used for ATMT transformation of the WT and Δ*MrBI-1* to generate the mutants WT::*Bax* and Δ*MrBI-1*::*Bax*, respectively. To confirm successful gene deletion and complementation, PCR and RT-PCR were performed using the primers MrBIF/MrBIR. To verify the successful expression of *Bax*, the fungi were grown in SDB for 3 d and used for RT-PCR analysis with primers BaxRT1 and BaxRT1 (Table 1). The 18S rRNA gene was amplified using primers 18SF and 18SR (Table 1) and used as an internal positive control^49^.

### Heat shock assay

Conidia of the WT, Δ*MrBI-1*, Comp, WT::*Bax* and Δ*MrBI-1*::*Bax* were collected in 0.05% (v/v) Tween-80 and the spore suspensions (1 ml, 2 × 10^7^ conidia/ml) were transferred into Eppendorf tubes for treatment at 45 °C in a water bath for different time periods. The aliquots of suspensions (10 μl) were inoculated onto PDA medium (3 ml in plastic Petri dishes, 6 cm diameter) and the plates were incubated at 25 °C for 24 h for examination of conidium survivals. There were three replicated plates per strain and the experiment was repeated twice.

### Conidial stress challenges and survival assays

For cell death analysis, WT germlings were treated with or without the apoptosis-inducing compound farnesol (FOH, Sigma, prepared in dimethyl sulfoxide to final concentrations of 25 or 50 μM) at 25 °C for 4 h^34^. The dyes Hoechst 33342 and PI (Beyotime Ltd.) were used to stain cell nuclei and necrotic cells, respectively^3^. Observations using fluorescence microscopy were made with an Olympus microscope (BX51-33P, Olympus). The effect of FOH on cell viability was assayed by inoculation of the spores of WT and different mutants in SDB with or without (control) 50 μM FOH for 16 h or H_2_O_2_ (1 or 2 mM) for 12 h at 25 °C to determine spore germination rates. There were three replicated plates per strain and the experiments were repeated twice.

### Insect bioassays

To investigate the effect of MrBI-1 on fungal virulence, insect bioassays were conducted against the newly emerged fifth instar silk worm, *Bombyx mori*. Conidia of the WT, Δ*MrBI-1* and Comp harvested from the PDA plates were applied topically by immersing the larvae for 30 seconds in an aqueous suspension containing 2 × 10^7^ conidia/ml. Each treatment had three replicates with 15 insects each, and the experiments were repeated twice. Mortality was recorded every 12 h. The values of median lethal time (LT_50_) were calculated for each strain by Kaplan-Meier analysis^50^.

## Additional Information

**How to cite this article**: Chen, Y. *et al.* The Bax inhibitor MrBI-1 regulates heat tolerance, apoptotic-like cell death, and virulence in *Metarhizium robertsii.* *Sci. Rep.* **5**, 10625; doi: 10.1038/srep10625 (2015).

## Figures and Tables

**Table 1:** Primers used in this study.

**Primers**	**Sequences (5′-3′)**	**Purpose of use**
BaxF	CCAAGCTTGGCAGTGATGGACGGGTC	For gene function assays in yeasts.
BaxR	GCGTCGACTCAGCCCATCTTCTTCCAG	
BclF	CGGAATTCCCGGAAGGATGGCGCAAGC	
BclR	CGGAATTCTCACTTGTGGCCCAGGTA	
AtBIF	CGGAATTCGCGATTCTCAAAGTCAAG	
AtBIR	CGGAATTCTCAGTTTCTCCTTTTCTT	
MrBI1	CGGAATTCCGTCGCTGCCACCTCCCACAC	
MrBI2	CGGAATTCCTAGTTGTTGGACTGGCTGTTCA	
MrBI1U	CGGGATCCGGGTCCCACTCGATAACTGA	For deletion and verification of *MrBI-1* (MrBI1U was also used for complementation of *MrBI-1*).
MrBI1L	ACTGGATCCATGTCCGGTAG	
MrBI2U	GCTCTAGAGCTCTTCCTCTCGCTCTTCA	
MrBI2L	GCTCTAGACGATACGCTAGGCCTTTCTG	
MrBIF	AAGGTCTCTTCGGACCACCT	
MrBIR	GACGATGACGGAGATGGAGT	
MrBIXh	CCGCTCGAGATGGCCACCAACACCAAGTAC	For complementation of *ΔMrBI-1*.
MaBIEI	CGGAATTCCTAGTTGTTGGACTGGCTGTTC	
BaxHd	GGGAATTCGGCAGTGATGGACGGGTC	For engineering of *Bax* in *M. robertsii*.
BaxSa	GGGAATTCTCAGCCCATCTTCTTCCAG	
BaxRT1	TCATCCAGGATCGAGCAG	For RT-PCR analysis of *Bax* gene.
BaxRT2	CCAGATGGTGAGTGAGGC	
18SF	CAGGGCTCTTTTGGGTCTTG	For RT-PCR internal control.
18SR	AGTTTCAGCCTTGCGACCAT	
